# The Biosynthetic Pathway of Major Avenanthramides in Oat

**DOI:** 10.3390/metabo9080163

**Published:** 2019-08-07

**Authors:** Zhiyong Li, Yi Chen, Dauenpen Meesapyodsuk, Xiao Qiu

**Affiliations:** Department of Food & Bioproduct Sciences, University of Saskatchewan, Saskatoon, SK S7N 5A8, Canada

**Keywords:** *Avena sativa*, avenanthramides, hydroxycinnamoyl-CoA:hydroxyanthranilate *N*-hydroxycinnamoyl transferase, caffeoyl-CoA *O*-methyltransferase, 4-coumarate-CoA ligase

## Abstract

Avenanthramides are a group of *N*-cinnamoylanthranilic acids, with health-promoting properties mainly found in oat (*Avena sativa* L.). However, the biosynthetic mechanism for the main three types of avenanthramides (Avn-A, Avn-B and Avn-C) is not completely understood. In the present study, we report molecular identification and functional characterization of three different types of genes from oat encoding 4-coumarate-CoA ligase (4CL), hydroxycinnamoyl-CoA:hydroxyanthranilate *N*-hydroxycinnamoyl transferase (HHT) and a caffeoyl-CoA *O*-methyltransferase (CCoAOMT) enzymes, all involved in the biosynthesis of these avenanthramides. In vitro enzymatic assays using the proteins expressed in *Escherichia coli* showed that oat 4CL could convert *p*-coumaric acid, caffeic acid and ferulic acid to their CoA thioesters. Oat HHTs were only responsible for the biosynthesis of Avn-A and Avn-C using hydroxyanthranilic acid as an acyl acceptor and *p*-coumaroyl-CoA and caffeoyl-CoA as an acyl donor, respectively. Avn-B was synthesized by a CCoAOMT enzyme through the methylation of Avn-C. Collectively, these results have elucidated the molecular mechanisms for the biosynthesis of three major avenanthramides in vitro and paved the way for metabolic engineering of the biosynthetic pathway in heterologous systems to produce nutraceutically important compounds and make possible genetic improvement of this nutritional trait in oat through marker-assisted breeding.

## 1. Introduction

The human food market for oat is increasingly important as the consumers gradually recognize its health benefits [1]. Oat is considered as a healthy grain primarily due to the presence of β-glucan, a mixed-linkage (β1→3, β1→4) glucose polymer that can reduce the risk of heart diseases by lowering blood cholesterol levels [2]. In addition, oat grain contains a higher level of nutritious storage proteins than other cereals, as it is the only cereal crop that contains avenalin as the major storage protein, a legumin-like globulin equivalent in nutritional quality to soybean proteins [3]. Furthermore, oat contains avenanthramides, a group of polyphenolic compounds that possess strong antioxidant, anti-inflammatory, and anti-cell-proliferating properties that have been linked to the prevention of cardiovascular diseases, and the protection of human skin [4,5,6,7].

Avenanthramides were first identified as phytoalexins in oat leaves infected by pathogenic fungus *Puccina coronata* [8], but they were later found at significant levels in oat grains [9,10]. Avenanthramides are a group of *N*-cinnamoylanthranilic acids comprising anthranilic acid and cinnamic acid linked by an amide bond [11]. Due to the presence of various substituted groups on the two components, more than 25 different types of avenanthramides have been detected in oat grains. However, the most abundant ones are three comprising 5-hydroxyanthranilic acid conjugated with caffeic acid (as avenanthramide-C or Avn-C), *p*-coumaric acid (as avenanthramide-A or Avn-A) or ferulic acid (as avenanthramide-B or Avn-B) [9].

Despite the health importance of avenanthramides, the biosynthesis of these compounds in oat has not been completely understood. Previous reports indicate that avenanthramides are synthesized through a condensation process of hydroxyanthranilic acid with hydroxycinnamoyl-CoA and related species, which is catalyzed by hydroxycinnamoyl-CoA:hydroxyanthranilate *N*-hydroxycinnamoyltransferase (HHT), an anthranilic acid acyl-CoA dependent acyltransferase. HHT is homologous to hydroxycinnamoyl-CoA:shikimate/quinate hydroxycinnamoyl transferase (HCT) catalyzing the coupling of *p*-coumaroyl-CoA with shikimate/quinate [12,13,14,15]. Both HHT and HCT belong to the BAHD acyltransferase family where two more acetyltransferases benzylalcohol *O*-acetyltransferase (BEAT) and deacetylvindoline 4-*O*-acetyltransferase (DAT) are also included to catalyze the formation of a diverse group of plant metabolites using CoA thioesters as acyl donors [14]. In particular, HCT is a sub-family of well-conserved enzymes among land plants participating in the biosynthesis of lignins and flavonoids that function by acylating a wide range of aroyl group-containing substrates to appropriate acceptors [13,16,17,18,19]. Therefore, acyl donors such as hydroxycinnamoyl-CoA and derivatives, precursors for the biosynthesis of avenanthramides are probably diverged from the phenylpropanoid pathway and their biosynthesis may require 4-coumarate: CoA ligase (4CL) for the activation of aroyl group-containing substrates to the corresponding thioesters for the subsequent condensation process [20,21,22]. In addition, the exact mechanism for the biosynthesis of Avn-B has not been conclusively determined, although this avenanthramide might be synthesized by the same HHT using *p*-coumaroyl-CoA and feruloyl-CoA as substrates [12,13]. The incomplete and inconclusive information on the biosynthesis of avenanthramides has hindered genetic improvement of this important nutritional trait in oat. 

In the present study, we report the identification and characterization of the genes involved in the biosynthesis of oat major avenanthramides. An oat 4CL converts hydroxy or methoxy cinnamic acid derivatives to their corresponding thioesters. Oat HHTs are involved in the biosynthesis of Avn-A and Avn-C by the condensation of hydroxyanthranilic acid with *p*-coumaroyl-CoA and caffeoyl-CoA, respectively. Avn-B is synthesized by methylation of the hydroxyl group at position 3 of the aroyl group in Avn-C by a CCoAOMT enzyme. Elucidation of the biosynthesis of the major avenanthramides would facilitate breeding efforts to improve this important nutritional trait by functional DNA markers-assistant breeding in oat.

## 2. Results

### 2.1. Identification and Functional Analysis of Genes Encoding 4CLs in the Biosynthesis of Hydroxycinnamate Thioesters

**Figure 1:**
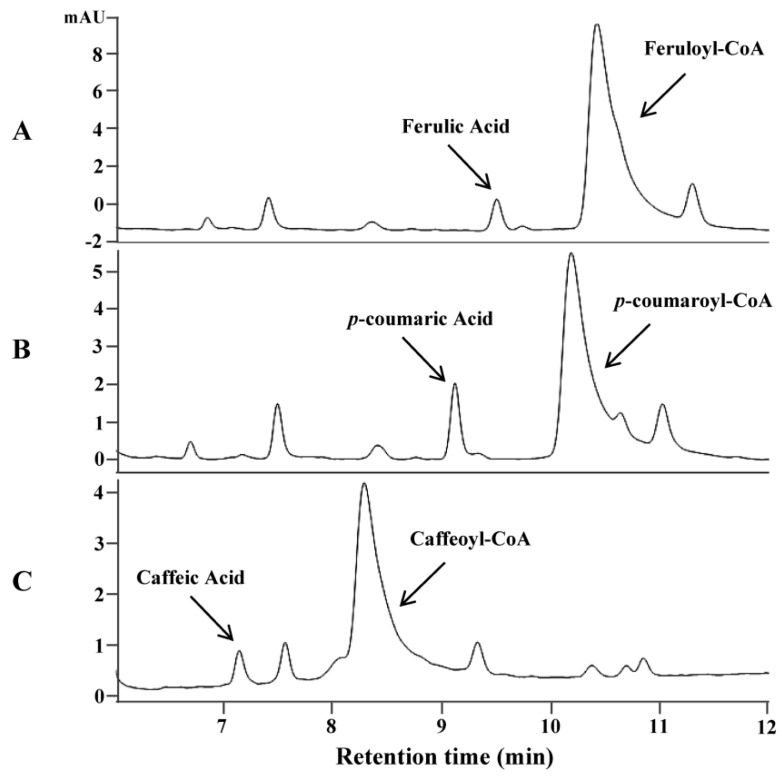
In vitro assays of oat 4CL gene (As4CL1) using the protein expressed in *E. coli* on three substrates. HPLC analysis of the products on substrate ferulic acid (**A**), *p*-coumaric acid (**B**), and caffeic acid (**C**).

Avenanthramides are the condensed products of anthranilic acid and hydroxycinnamic acid using hydroxycinnamoyl-CoA as an acyl donor and anthranilate as an acyl acceptor. The biosynthesis of hydroxycinnamoyl-CoAs is generally believed to be catalyzed by 4-coumarate-CoA ligase (4CL) converting hydroxycinnamic acids to corresponding thioesters [23]. To identify genes encoding this enzyme in oat, an *Arabidopsis thaliana* 4CL was used as a query to search a transcriptome database of oat developing seeds [24]. Two candidate genes (As4CL1 and As4CL2) coding for putative 4CL were identified. Sequence analysis of these two genes revealed that they shared high sequence identity with each another throughout the open reading frame (ORF), except for the middle region where an insertion of a few nucleotides occurred in As4CL2, which resulted in the considerable difference in amino acid sequences of the region (Supplementary Figure S1). As4CL1 was much close to putative 4CL enzymes from other grass plants and further chosen for enzyme assay. Functional analysis of As4CL1 by in vitro assays using the purified protein expressed in *E. coli* showed that the enzyme could convert three substrates, ferulic acid, *p*-coumaric acid and caffeic acid, to their corresponding thioesters. Under the assay conditions with one hour reaction time and the same concentration of substrates, over 90% of ferulic acid, 62% of *p*-coumaric acid and 52% of caffeic acid were converted to their corresponding CoA thioesters, respectively (Figure 1). On the contrary, no product was found by boiled As4CL1 proteins with corresponding precursors (Supplementary Figure S2).

### 2.2. Identification and Functional Analysis of New Genes Encoding HHT in the Biosynthesis of Avn-A and Avn-C

**Figure 2:**
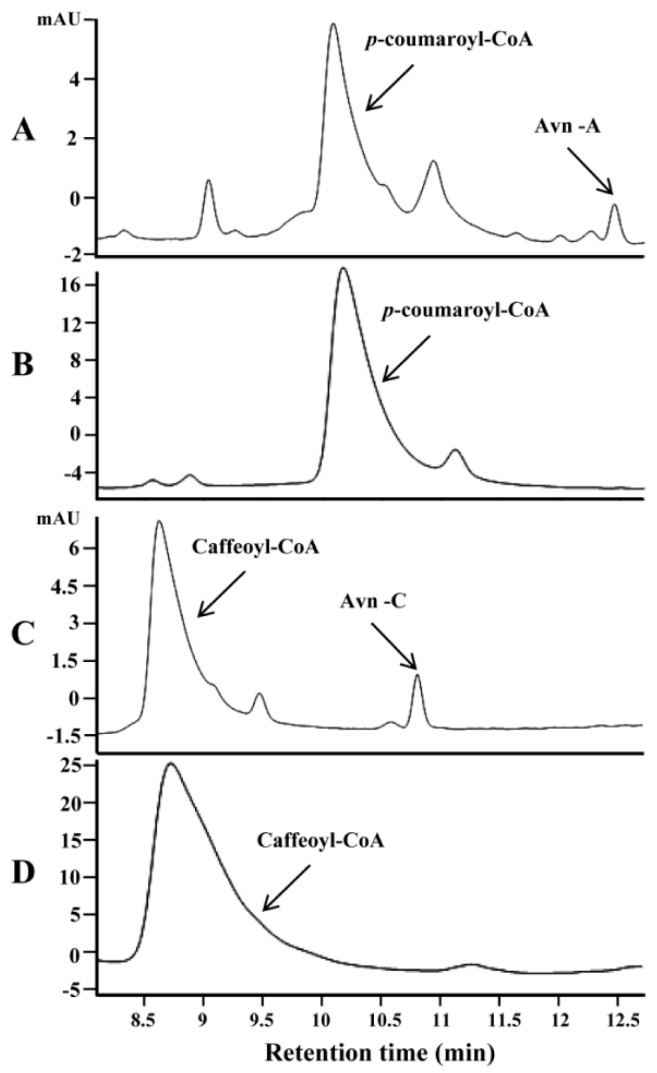
In vitro assays of oat HHT gene (AsHHT1) using the protein expressed in *E. coli*. HPLC analysis of the products in the presence of 5-hydroxy-anthranilic acid with *p*-coumaroyl-CoA (**A**) and with caffeoyl-CoA (**C**). The negative controls with boiled AsHHT enzymes were shown in (**B**,**D**) with corresponding substrates.

Three genes encoding HHT (AsHHT1-3) in the biosynthesis of avenanthramides were previously identified in oat [13]. Encoded isozymes AsHHT1-3 shared very high amino acid identity (from 95% to 98%). By searching the oat seed transcriptome [24] using these sequences as queries, three new HHT genes (AsHHT4-6) were identified that shared high sequence identity with each other (about 95%) but were somewhat more distinct to AsHTT1-3 sharing about 82% amino acid identity with them (Supplementary Figure S3). Representing each group, AsHHT1 and AsHHT4 were expressed in *E. coli*. Both purified enzymes could catalyze the condensation of 5-hydroxyanthranilic acid and *p*-coumaroyl-CoA, producing a product with the retention time identical to standard Avn-A (as shown in Figure 2A for AsHHT1). Mass spectrum analysis confirmed the authenticity of the product on the basis of the molecular ion at *m*/*z* 298.1 and a fragment at *m*/*z* 254.0 yielded by the removal of carboxylic group (Supplementary Figure S4A). In addition, both enzymes could also condense 5-hydroxyanthranilic acid and caffeoyl-CoA to a product with the retention time and mass spectra identical to standard Avn-C (Figure 2C, and Supplementary Figure S4B). In contrast, no HHT activity was detected with boiled enzymes (Figure 2B,D). Surprisingly, when feruloyl-CoA and 5-hydroxyanthranilic acid were supplied, no new product was produced by the two enzymes, indicating AsHHTs were incapable of synthesizing Avn-B by condensation of the two perceived substrates (data not shown). 

### 2.3. Identification and Functional Analysis of a Gene Encoding a CCoAOMT Enzyme in the Biosynthesis of Avn-B

**Figure 3:**
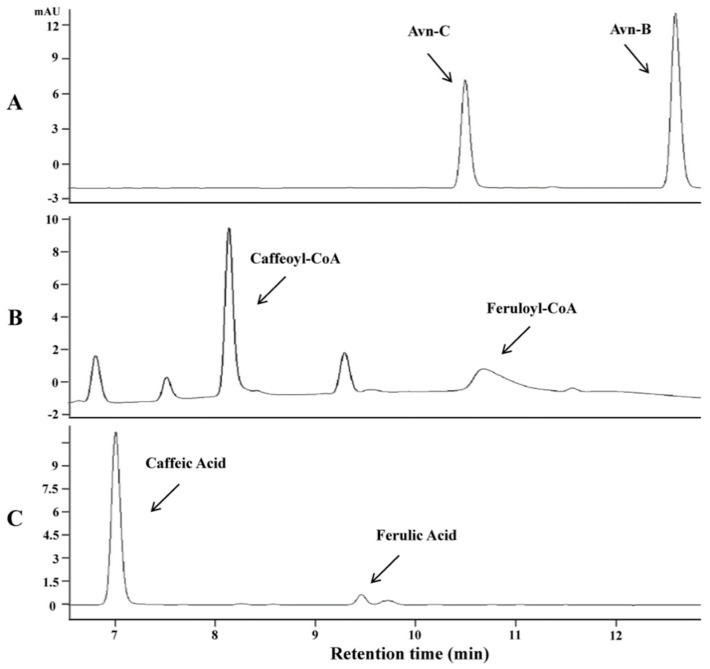
In vitro assays of CCoAOMT using the protein expressed in *E. coli* on three substrates. HPLC analysis of the products in the presence of S-adenosyl methionine with Avn-C (**A**), caffeoyl-CoA (**B**) and caffeic acid (**C**).

As AsHHTs appeared not involved in the biosynthesis of Avn-B, we hypothesized that this avenanthramide might be synthesized by a different mechanism. A previous study showed that one of the major metabolites in mice fed with Avn-C was identified as Avn-B [25]. As caffeoyl-CoA *O*-methyltransferases could methylate a wide range of substrates, we assumed that this type of *O*-methyltransferases might be able to convert Avn-C to Avn-B by methylation. To test the hypothesis, we identified a single CCoAOMT gene from the oat transcriptome database [24] with an ORF) 768 nucleotides encoding 256 amino acids. In vitro assays of this gene using the purified protein expressed in *E. coli* in the presence of S-adenosyl methionine showed that the enzyme indeed could convert Avn-C to a product with retention time and mass spectra identical to Avn-B (Figure 3A). To examine the substrate specificity, three possible substrates, Avn-C, caffeoyl-CoA and caffeic acid, were employed for kinetic analysis, which showed that the CCoAOMT enzyme exhibited activity towards all three substrates tested. However, the most preferred substrate was Avn-C compared to caffeoyl-CoA and caffeic acid. The ratio of Vmax to Km was 3.08 with Avn-C, five or ten times higher than those with caffeoyl-CoA and caffeic acid (0.70 and 0.31), respectively (Table 1, and Supplementary Figure S5). These results clearly indicate that Avn-B can be synthesized from Avn-C through the methylation process catalyzed by this CCoAOMT enzyme.

**Figure 4:**
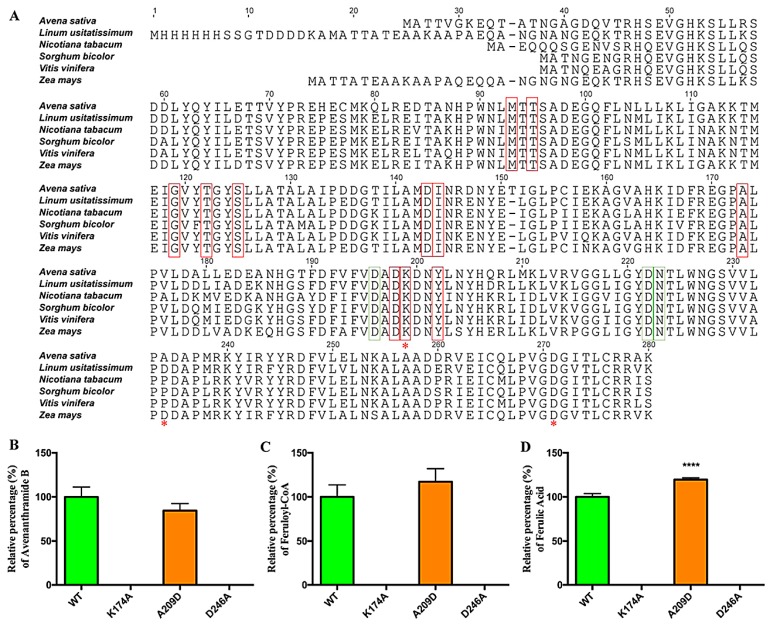
Mutagenesis analysis of the oat CCoAOMT enzyme. (**A**) Sequence alignment of oat CCoAOMT sequence and related sequences. Conserved residues involved in SAM binding were highlighted in red boxes. Residues involved in divalent binding were highlighted in green boxes. The loop region was between N227 to L250. Mutation site were marked by red *. Changes in the activity of native and mutagenized CCoAOMT proteins on three substrates avenanthramide C (**B**), caffeoyl-CoA (**C**), and caffeic acid (**D**) were calculated using the average of three biological triplicate measurements.

CCoAOMTs were identified and functionally analyzed from a variety of plants species. They were highly conserved in residues involved in the binding of *S*-Adenosyl-L-methionine (SAM), and metal ion. Two residues proximal to the active site, lysine and aspartic acid located in the C-terminal region, are involved in substrate binding and catalysis [26]. In addition, a loop structure at the C-terminus is probably involved in the recognition of substrates [27]. To confirm the importance of these residues in the oat CCoAOMT enzyme, two conserved residues at the presumed active sites (K174, D246) and one residue (A209) in the loop region possibly responsible for substrate recognition were mutagenized (Figure 4A). Neither mutation impacted heterologous expression in *E. coli* (Figure S6), but mutation of either lysine at 174 or aspartic acid at 246 to alanine completely abolished enzyme activity towards all three substrates (Figure 4B–D), which coincides with the previous result that these two residues are likely involved in the catalysis [26]. The mutation of alanine 209 to aspartic acid, the corresponding residue in a sorghum CCoAOMT with substrate specificity to caffeoyl-CoA, seemed to decrease the activity towards Avn-C, and increase the activity towards caffeoyl-CoA and caffeic acid slightly. However, this change was not statistically significant.

## 3. Discussions

Avenanthramides are a group of phenolic compounds found almost exclusively in oat. Three major avenanthramides in oat are conjugates of hydroxycinnamic acid; *p*-coumaroyl-CoA (Avn-A), feruloyl-CoA (Avn-B) and caffeoyl-CoA (Avn-C). The biosynthesis of these compounds was previously believed to be catalyzed by a single enzyme called hydroxycinnamoyl CoA:hydroxyanthranilate *N*-hydroxycinnamoyl transferase (HHT) by the condensation of hydroxyanthranilate and substituted cinnamoyl-CoA thioesters [12,13]. However, our enzymatic assays with two oat HHT proteins demonstrated that the enzymes catalyzed the N-acylation of 5-hydroxyanthranilic acid with *p*-coumaroyl-CoA or caffeoyl-CoA, but not with feruloyl-CoA, indicating that oat HHTs are only involved in the biosynthesis of Avn-A and Avn-C, but not Avn-B. A previous in vitro assay using crude protein extracts from oat showed that Avn-B was produced in the presence of 5-hydroxyanthranilic acid and feruloyl-CoA [12]. As oat crude protein extracts contain numerous enzymes, this result could arise from other enzymatic activities in the extract. In addition, when oat AsHHT1 was expressed in *E. coli*, in vitro assays using the crude protein extracts detected a low level of activity for the synthesis of Avn-B in the presence of 5-hydroxyanthranilic acid and feruloyl-CoA [13]. However, our in vitro assays using purified proteins of AsHHT1 and AsHHT4 expressed in *E. coli* showed that both could not catalyze the condensation of 5-hydroxyanthranilic acid and feruloyl-CoA, giving rise to Avn-B. The reason why the different results were obtained by the two experiments on the same gene is currently unknown. The possible explanation is that the low level of activity might also be derived from other factors in the crude proteins from the expression host in the previous experiment. 

As oat HHTs are only responsible for the synthesis of Avn-A and Avn-C, the next question would be identifying the biosynthetic mechanism for Avn-B. In consideration of the structural difference of Avn-C and Avn-B, we assumed that certain CCoAOMT would be able to convert Avn-C to Avn-B by methylation as this enzyme is known to have a wide range of substrates. In fact, a previous feeding study showed that one of the major metabolites from Avn-C in mice was Avn-B [25]. Indeed, when a CCoAOMT enzyme from oat was expressed in *E. coli*, the purified protein was capable of the synthesis of Avn-B on Avn-C in the presence of *S*-adenosyl methionine, indicating that Avn-B is synthesized by the *O*-methylation of Avn-C catalyzed by this CCoAOMT enzyme. 

In plants, there are two types of *O*-methyltransferases responsible for methylating hydroxyl groups at the 3- and 5-positions of a phenolic ring. The first type called caffeic acid *O*-methyltransferase (COMT) is larger in size (38–43 kD) with methylation activity mainly on caffeic acid and related species [28,29], while the second type called caffeoyl-CoA *O*-methyltransferase (CCoAOMT) is smaller in size (23–27 kD) and has methylation activity mainly for caffeoyl-CoA and its derivatives [30,31]. The oat O-methyltransferase with Avn-B synthetic activity belongs to the type II O-methyltransferase (CCoAOMT) [30,31]. The residues involved in the binding of SAM and a metal ion, and proximal to the active site for catalysis [26], and the loop structure for the recognition of substrates were highly conserved [27]. In our assays, mutations of two conserved residues lysine and aspartic acid proximal to the active site to alanine completely abolished the catalytic activity of the oat CCoAOMT enzyme towards all substrates tested, consistent with an involvement of these residues in the catalysis [26]. However, substitution of alanine at position 209 with the corresponding residue aspartic acid in a sorghum CCoAOMT with substrate specificity to caffeoyl-CoA in the loop did not significantly alter activity towards Avn-C and caffeoyl-CoA, indicating that this amino acid in the loop might not play a vital role in defining its substrate specificity [29].

**Figure 5:**
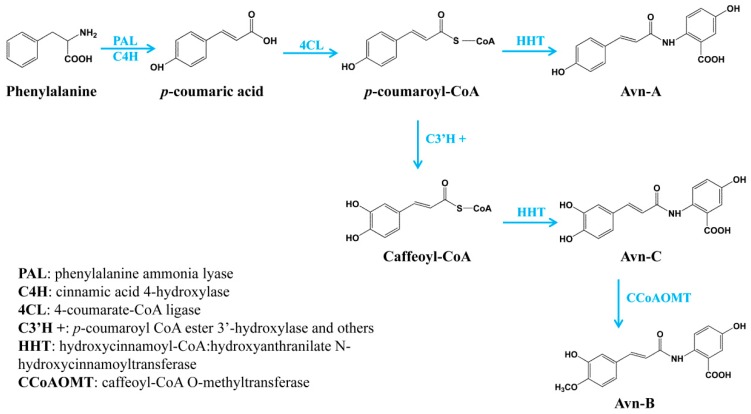
The complete biosynthetic pathway of three major avenanthramides in oat.

Oat has been considered as a functional food with many health benefits. One of the primary effective ingredients in oat grain is avenanthramides, a group of polyphenolic compounds with antioxidant, anti-inflammatory, anti-cell-proliferating and skin anti-irritant properties. Although the health-promoting properties of avenanthramides are well known, the biosynthetic mechanism was not completely understood. In the present study, we identified three different types of enzymes involved in the biosynthesis of the major avenanthramides in oat: 4CL in activating hydroxycinnamates to their thioesters prior to the condensation, HHTs catalyzing the condensation in the biosynthesis of Avn-A and Avn-C, and CCoAOMT enzyme for the methylation of Avn-C to Avn-B. Particularly, we demonstrated that oat HHTs are only responsible for the biosynthesis of Avn-A and Avn-C, but not for Avn-B, which is synthesized by a new mechanism, the methylation of Avn-C catalyzed by CCoAOMT enzyme. For the complete biosynthesis of the three major avenanthramides in oat, *p*-coumaric acid is initially derived from phenylalanine catalyzed by phenylalanine ammonia lyase (PAL) and cinnamic acid 4-hydroxylase (C4′H). *p*-coumaric acid can be activated into its CoA thioesters by 4CL, which can then be condensed with 5-hydroxyanthranilic acid to Avn-A by HHT. On the other hand, *p*-coumaroyl-CoA is often converted to *p*-coumaroyl shikimate/quinate first, which is then possibly hydroxylated by *p*-coumaroyl CoA ester 3′-hydroxylase (C3′H), a cytochrome P450 enzyme (CYP98) [32,33]. Caffeoyl-CoA can then be condensed with 5-hydroxyanthranilic acid to Avn-C by HHT. Finally, Avn-C is methylated to Avn-B by CCoAOMT enzyme (Figure 5). Taken together, the full elucidation of the biosynthetic pathway of avenanthramides in the present study not only contributes to our understanding of the biosynthesis of these important nutraceutical compounds, but also facilitates genetic improvement of this nutritional trait in oat by marker-assisted breeding and open opportunities to produce these active compounds by metabolic engineering of the complete biosynthetic pathway in heterologous systems.

## 4. Materials and Methods 

### 4.1. Plant Materials

Oat (*Avena sativa* L.) cultivar ‘CDC Dancer’ (Crop Development Centre, University of Saskatchewan, Canada) seeds were sterilized with 10% (*w*/*v*) NaClO for 5 min, washed with sterilized water 3 times, and germinated on filter paper with water in a plate in the dark at room temperature. After several days, the germinated seeds were transferred into the soil and grown in a controlled growth chamber at 22 °C with 65% relative humidity under 16 h light and 8 h dark cycles.

### 4.2. HPLC Analysis

HPLC analysis of avenanthramides was carried out on an Agilent 1100 high pressure liquid chromatography (HPLC) instrument equipped with a 3 µm, 4.6 × 150 mm Phenomenex Luna C18 column (Torrance, CA, USA) and an Agilent photodiode array detector (Santa Clara, CA, USA) at 340 nm. The mobile phase consisted of solvent A (H_2_O with 5% acetonitrile and 0.1% formic acid) and solvent B (acetonitrile with 0.1% formic acid). A gradient of 13% to 30% buffer B over 20 min at a flow rate of 1.0 mL/min was applied. The avenanthramides were identified by comparison of chromatography retention times and mass spectra to authentic standards (avenanthramide A, B and C) (Sigma, St. Louis, MO, USA). Mass spectrometry analysis was performed following [34].

### 4.3. RNA Extraction, cDNA Synthesis and Genes Cloning

RNA extraction from developing seeds at about 20 days after pollination and cDNA synthesis were performed following the methods from our previous study [35]. To clone candidate genes, primers were designed to amplify the full open reading frames by PCR with Q5 polymerase (NEB, Ipswich, MA, USA). For AsHHT4-6 cloning, the two primers were HHT4F (*EcoR*I): 5′-ACTGAATTCATGCACGGTGAGGCGGTC-3′ and HHT4R (*Hind*III): 5′-AAGCTTCAGCCTGCTCACACGTCGGCGATCAG-3′. For oat 4CL cloning, the two primers were 4CL1F (*BamH*I): 5′-GGATCCAGATCGATGGGCTCCATCG-3′ and 4CL1R (*Xho*I): 5′-CTCGAGCTGACTTAGCTTTTGGACTGTG-3′. For oat CCoAOMT cloning, the two primers were CCoAOMT1F (*EcoR*I): 5′-CGGAATTCATGGCGACCACGGC-3′ and CCoAOMT1R (*Hind*III): 5′-CGAAGCTTTCACTTGGCGCG-3′. The PCR products were cloned into the pGEM vector (Promega, Madison, WI, USA) and verified by sequencing. For the construction of plasmids in the expression in *E. coli*, verified fragments were released from pGEM and sub-cloned into the destination vector pET-28a with specific restriction enzymes highlighted in the primers. For site-directed mutagenesis in CCoAOMT, three sets of primer were applied: K174A-F (5′-CGACGCCGACGCCGACAACTACC-3′) and K174A-R (5′-GGTAGTTGTCGGCGTCGGCGTCG-3′); A209D-F (5′-GTGCTCCCCGACGACGCGCCC-3′) and A209D-R (5′-GGGCGCGTCGTCGGGGAGCAC-3′); D246A-F (5′-CCCGTCGGAGCCGGCATCAC-3′) and D246A-R (5′-GTGATGCCGGCTCCGACGGG-3′). Site-directed mutagenesis was performed using an overlapping PCR in two steps. The first PCR was performed to amplify two fragments surrounding a mutation using the native gene sequence as template and two sets of primers: T7 promoter-F and K174A-R, and K174A-F and T7 terminator-R. The second PCR was performed using the mixture of the two fragments as template and one set of primers: T7 promoter-F and T7 terminator-R. The amplified product was digested with *EcoR*I and *Hind*III and sub-cloned into pET-28a vector at the same digested sites for expression analysis. The expression plasmids were verified by restriction enzyme digestion and sequencing.

### 4.4. Protein Expression and Purification

The pET-28a recombinant constructs were introduced into *E. coli* BL21 (DE3) (Novagen, CA, USA) for protein expression. Expression was induced by the addition of 0.5 mM isopropyl β-D-1-thiogalactopyranoside (IPTG) to cultures (A_600_ at 0.4 to 0.5), and the induced cells were incubated overnight at 16 °C. The cells were harvested by centrifugation at 5000× *g* for 10 min. The pellets were resuspended in a buffer containing 100 mM Tris/HCl, pH 8.0, 0.5 M NaCl, 20 mM imidazole and 5% glycerol. Cells were disrupted by glass beads using a Mini-Beadbeater, and lysate was subsequently centrifuged at 15,000× *g* for 15 min at 4 °C. The recombinant His-tagged fusion proteins were purified by Hispur Ni-NTA Resin (Thermo Fisher Scientific, Waltham, MA, USA) according to the manual. The purified proteins were further desalted and concentrated with Zeba Spin Desalting Columns (Thermo Fisher Scientific, Waltham, MA, USA). Concentration for purified protein samples was determined using a Bradford assay.

### 4.5. Enzyme Assays

Enzymatic assays of oat 4CL were performed following the previous method with some modification [36]. The reaction took place in a MOPS buffer (100 mM, pH 7.5) in a total volume of 300 μL consisting of 0.4 mM substrate (*p*-coumaric acid, caffeic acid, or ferulic acid), 2.5 mM ATP, 2.5 mM MgCl_2_, 0.2 mM Coenzyme A, 1~10 μg of purified oat 4CL proteins. Enzymatic reactions were initiated by the addition of purified 4CL protein. The reaction with boiled (96 °C, 10 min) proteins was used as the control. The assay was performed at 30 °C and formation of CoA esters was monitored using a UV spectrophotometer at 333 nm for *p*-coumaroyl-CoA, 346 nm for caffeoyl-CoA and feruloyl-CoA in a time course until the substrate conversion to CoA esters was complete. Assays were terminated by adding 10 μL of acetic acid and analyzed by HPLC with the same conditions as above. CoA-thioester standards were obtained from Microcombichem (Germany). The identity of products was determined based on their retention times and UV-visible absorbance spectral profiles compared with those of the standards. The relative conversion efficiency was calculated by product/(product + substrate). 

Oat HHT activity was determined by reacting 10 μL of the purified protein extracts (about 10 μg) with 100 mM 5-hydroxy-anthranilic acid in DMSO and 100 mM one of *p*-coumaroyl-/caffeoyl-/feruloyl-CoA at 30 °C for 1 h in 30 mM Tris-HCl (pH 7.2) in a total reaction volume of 100 μL. The reaction was initiated by adding the protein and stopped by adding 20 μL acetic acid. The reaction mixture was diluted with 0.38 mL methanol, filtered through 0.22 μm filter and analyzed by HPLC. Mass spectrum analysis of products in the assays was performed following a method described in a previous study [37].

The assay of oat CCoAOMT protein was performed following a previous study with modifications [31]. The reaction was comprised of 10 μg purified protein, 1 mM oversaturated SAM (Sigma, USA), 0 to 2 mM Avenanthramide C, 0 to 5 mM caffeoyl-CoA, 0 to 10 mM caffeic acid (Sigma, USA), 50 mM Tris- HCl, 0.2 mM MgCl_2_, 2 mM DTT, 10% glycerol, and 0.2 mM PMSF, which was incubated at 25 °C for 5–30 min. For the kinetic analysis, product formations relative to substrate concentrations were determined under the optimized condition. Product formation was quantified using HPLC as described above. Velocities were calculated as the amount of products formed (in μmol) based on the peak area compared to a standard curve per amount enzyme used (in μg) and time incubated (in min). Km and Vmax parameters were calculated using non-linear regression to the Michaelis-Menten kinetics using Prism 6.10 software (Graphpad Software, Inc., San Diego, CA, USA). 

## Figures and Tables

**Table 1:** Kinetic parameters of oat CCoAOMT enzyme with three substrates.

Substrate	Km (μM)	Vmax (nmol/μg/min)	Vmax/Km (nmol/μg/mim/μM)
Avenanthramide C	167.0 ± 25.2	514.4 ± 41.0	3.08
Caffeoyl-CoA	2319.0 ± 867.6	1620.0 ± 390.0	0.70
Caffeic Acid	1903.0 ± 173.5	597.6 ± 40.0	0.31

## Supplementary Materials

The following are available online at https://www.mdpi.com/2218-1989/9/8/163/s1, Figure S1. Sequence alignment for two 4CLs from oat with the coding region sequence (CDS) (**A**) and amino acids (**B**). The shaded regions indicate amino acids that are identical to each other. The two conserved domains for 4CL enzymes were shown in red box; Figure S2. The negative controls with boiled As4CL1 enzymes were shown with corresponding subtracts. Figure S3. Full sequences alignment of oat HHTs. Shaded regions indicate amino acids that are identical to each other. Black double-headed arrows indicate the conserved domain HXXXDG and motif DFGWG in BAHD acyltransferases; Figure S4. Representative images for the MS spectra of group fragments from the products Avn-A (**A**) and Avn-C (**B**). Putative dissociation mechanisms for these compounds were shown; Figure S5. Kinetic analysis of oat CCoAOMT enzyme. The kinetic constants were estimated from Michaelis-Menten plot using the average of three biological triplicate measurements; Figure S6. Representative image showing purified CCoAOMT proteins separated by 12% SDS-PAGE. M: prestained protein marker; A209D: CCoAOMT^A209D^; K174A: CCoAOMT^K174A^; D246A: CCoAOMT^D246A^; Nucleotide sequence data reported has been deposited in the GenBank under accession numbers: MH397063 (As4CL1), MH397064 (AsHHT4), MH397065 (AsHHT5), MH397066 (AsHHT6), and MK577959 (CCoAOMT).
