# Supplementary figures and images for: The Biosynthetic Pathway of Major Avenanthramides in Oat
Source: Metabolites. 2019 Aug 7;9(8):163. doi: 10.3390/metabo9080163 (PMC6724135; doi:10.3390/metabo9080163)

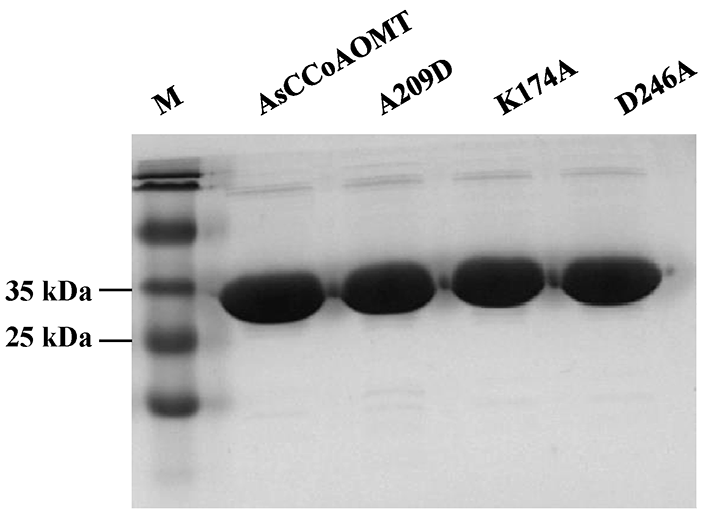

Supplement: Supplementary file 1 [file metabolites-09-00163-s001.zip › Figure S6.TIF]

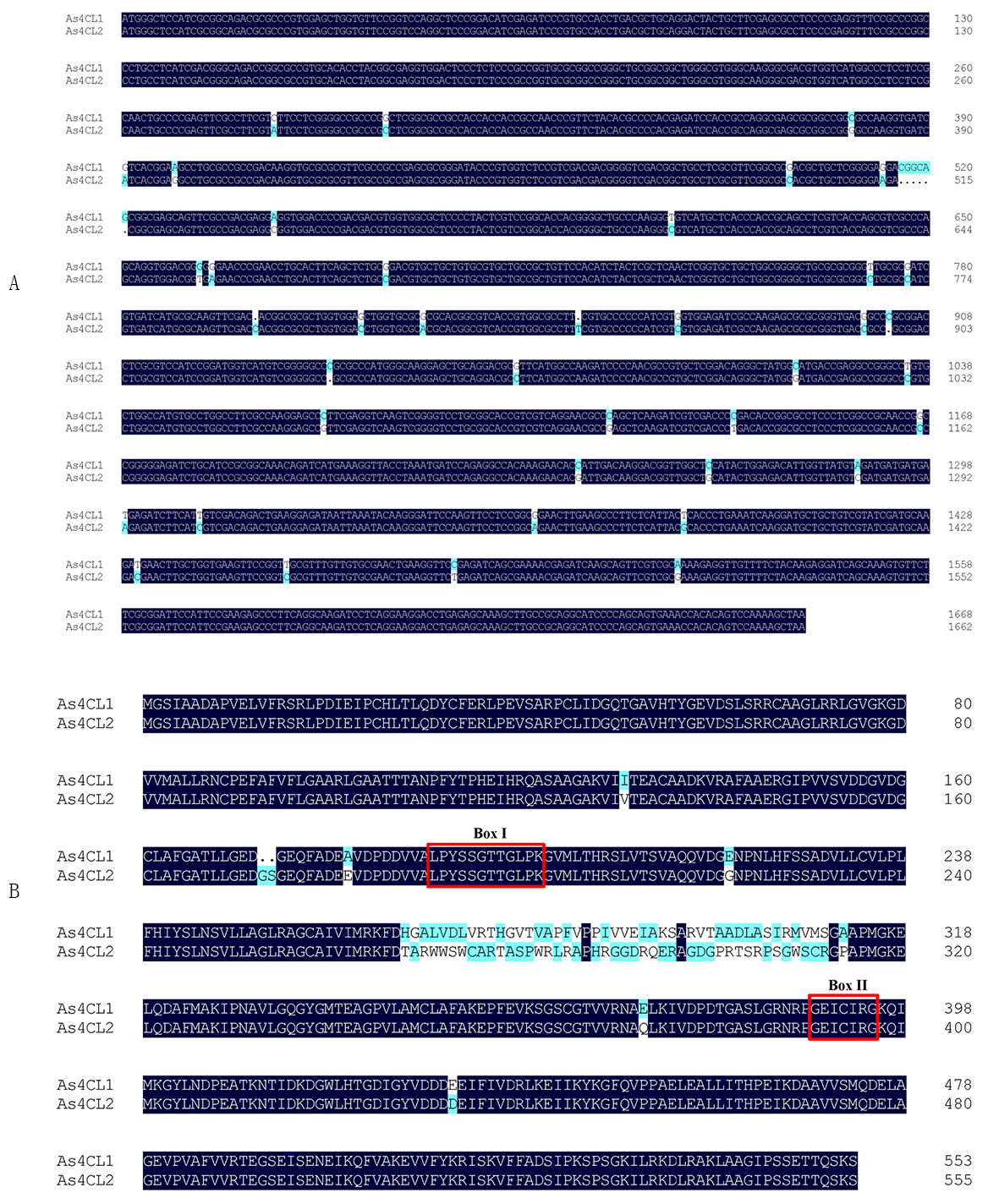

Supplement: Supplementary file 1 [file metabolites-09-00163-s001.zip › Figure S1.TIF]

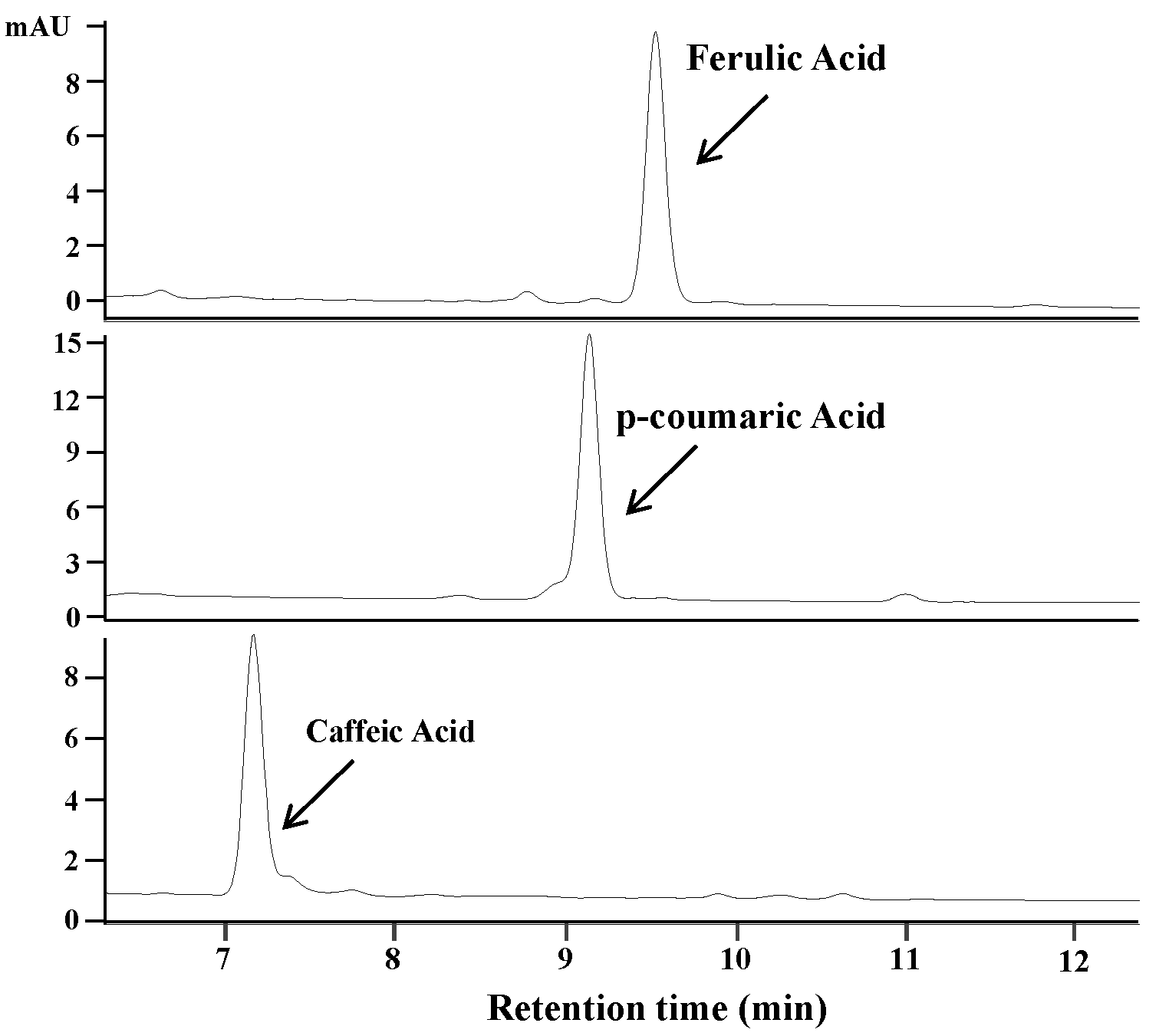

Supplement: Supplementary file 1 [file metabolites-09-00163-s001.zip › Figure S2.tif]

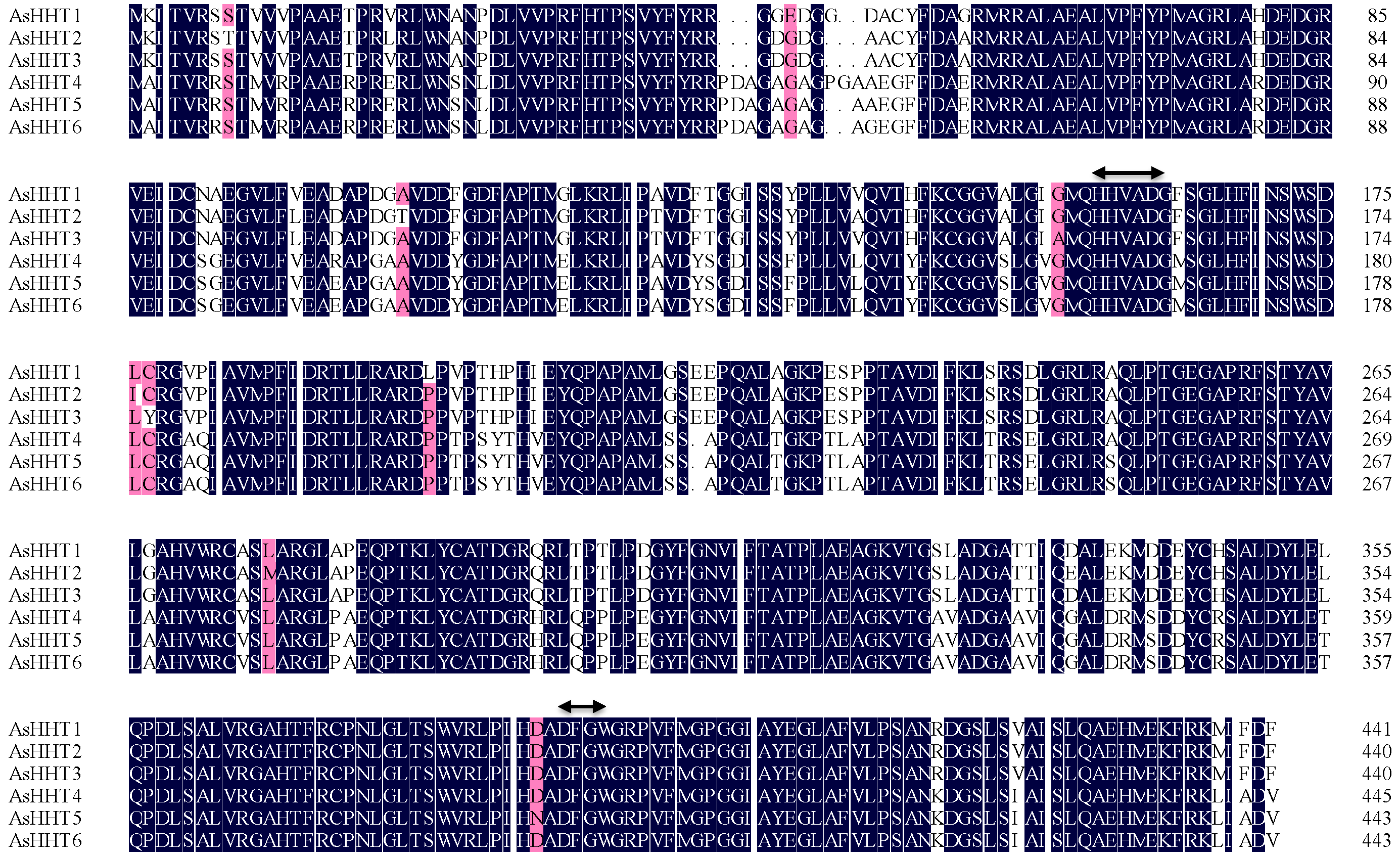

Supplement: Supplementary file 1 [file metabolites-09-00163-s001.zip › Figure S3.tif]

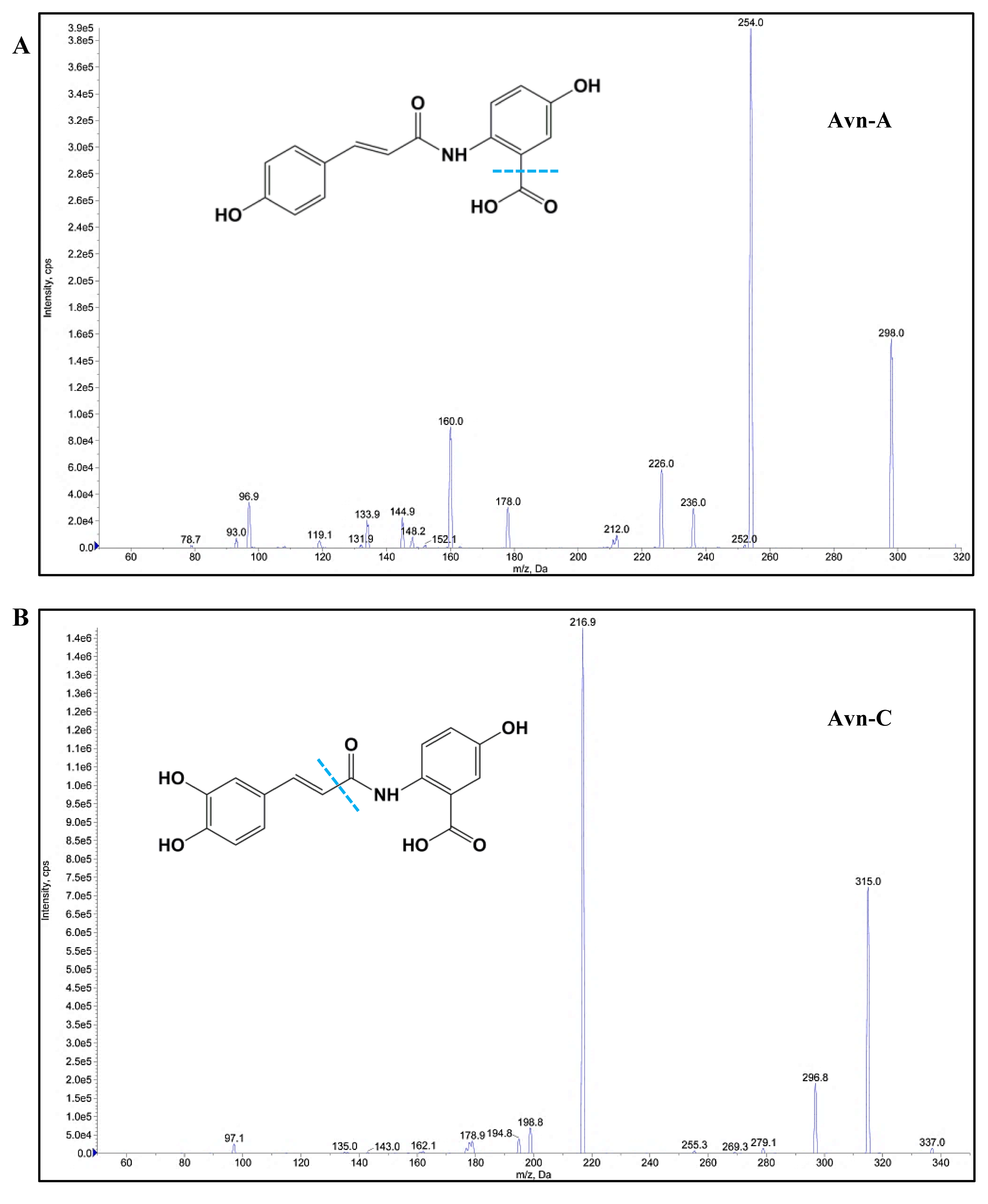

Supplement: Supplementary file 1 [file metabolites-09-00163-s001.zip › Figure S4.tif]

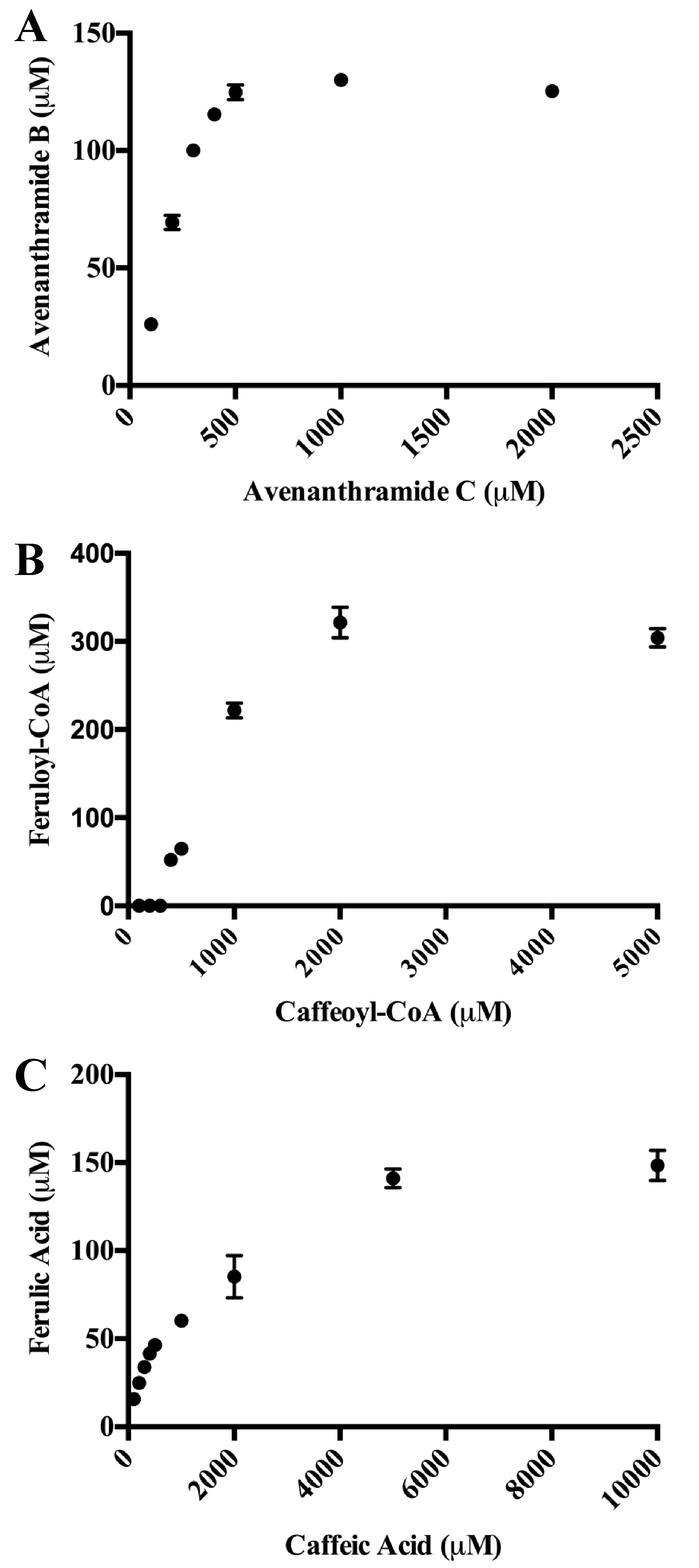

Supplement: Supplementary file 1 [file metabolites-09-00163-s001.zip › Figure S5.tif]
